# Childhood abdominal pain in primary care: design and patient selection of the HONEUR abdominal pain cohort

**DOI:** 10.1186/1471-2296-11-27

**Published:** 2010-04-08

**Authors:** Leo AA Spee, Arjan PJM van den Hurk, Yvonne van Leeuwen, Marc A Benninga, Sita MA Bierma-Zeinstra, Jan Passchier, Marjolein Y Berger

**Affiliations:** 1 Department of General Practice, Erasmus MC University Medical Center, Rotterdam, the Netherlands; 2 Department of Pediatric Gastroenterology, Emma Children's Hospital/Academic Medical Center, Amsterdam, the Netherlands; 3 Department of Medical Psychology and Psychotherapy, Erasmus MC University Medical Center, Rotterdam, the Netherlands; 4 Department of Clinical Psychology, VU University of Amsterdam, Amsterdam, the Netherlands

## Abstract

**Background:**

Abdominal pain in children is a common complaint presented to the GP. However, the prognosis and prognostic factors of childhood abdominal pain are almost exclusively studied in referred children. This cohort study aims at describing prognosis and prognostic factors of childhood abdominal pain in primary care. In this paper we describe methods used for data-collection and determine possible selective recruitment.

**Methods/Design:**

We conducted an observational, prospective cohort study with a 1-year follow-up. From May 2004 to March 2006, 53 Dutch GPs recruited consecutive children aged 4-17 years with a new episode of abdominal pain not preceded by a consultation for this complaint in the previous 3 months. Participants filled in standardized questionnaires, and faeces and urine were sampled. To evaluate selective recruitment, the electronic medical records of participating GPs were retrospectively searched for eligible non-included children.

**Discussion:**

This study allows us to describe prognosis and prognostic factors of childhood abdominal pain in primary care. A total of 305 children were included of whom 142 (46.6%) met predefined criteria for chronic/recurrent abdominal pain at presentation; from the total group of eligible children identified from the electronic medical record, 27% were included. The included children were significantly younger than non-included children (mean age 8.49 and 9.20 years). In proportion to identified eligible children, significantly less children diagnosed with "gastroenteritis" (6.8%) and significantly more children with "generalized abdominal pain" (39%) were included compared to the 27% that was expected. This cohort represents young school-aged children consulting GPs for a new episode of abdominal pain, not diagnosed as gastroenteritis. Almost half of them fulfil the criteria for chronic abdominal pain at presentation.

## Background

Abdominal pain is a frequent reason to consult the primary care physician. The prevalence of recurrent abdominal pain in school-aged children in Western countries ranges from 0.3 to 19% [1]. In the Netherlands abdominal pain in children is responsible for 2.5% of all childhood consultations in primary care and has a one-year prevalence rate of 63 per 1000 registered children [2]. About 25% of these children will visit their general practitioner (GP) more than once a year for this complaint. In 80% of the children the GP finally diagnoses the pain as "functional abdominal pain". The landmark study by Apley and Naish showed that in 90% of these children no organic cause could be identified [3]. When the abdominal pain becomes chronic it can have considerable impact on the child's well-being and the healthcare system [1,4]. A recent systematic review of prospective follow-up studies in children with chronic abdominal pain (CAP) at presentation showed that almost one third of children continue to have abdominal pain for more than 5 years, emphasizing the burden of disease [5]. Moreover, a relationship between childhood CAP and irritable bowel syndrome (IBS) in adulthood has been suggested [4,6-8].

Knowledge about determinants of the clinical course is essential for making management decisions and to inform patients and their parents about prognosis. Age, environmental factors, cultural background and psychosocial factors are often linked with the prognosis of childhood abdominal pain [9-12]. The prospective follow-up studies available suggest that parental factors rather than psychological characteristics of the child, predict the persistence of abdominal pain [13]; however, these studies are almost exclusively performed in referred children.

Therefore, to expand the information available to GPs, this cohort study was designed to collect data on prognosis and prognostic factors of childhood abdominal pain presented in primary care. In this current paper we describe methods used for data-collection and determine possible selective recruitment of our cohort.

## Methods/Design

This is a prospective, observational cohort study with a 1-year follow-up. Data were collected using standardized questionnaires, electronic medical records (EMRs), faeces and urine samples, and body mass index (BMI) was determined by measuring weight and height. All data collected during the study were documented anonymously and were not available for the GP. Researchers did not interfere with usual GP care. The study was approved by the Central Committee on Research Involving Human Subjects (CCMO) in the Netherlands.

### Patients

All patients aged 4-17 years consulting their GP for a new episode of acute or chronic abdominal pain, defined as a consultation for abdominal pain not preceded by a consultation for this complaint in the previous 3 months, were invited to participate in the study. Exclusion criteria were a previous diagnosis of inflammatory bowel disease, celiac disease or lactose intolerance, and inability to complete questionnaires due to language or cognitive problems.

### Setting and procedures

Twenty-five computerized general medical practices, including 53 GPs from the southwest region of the Netherlands, participated in the study. These medical practices represent a total population of 110,000 registered patients of which approximately 16,000 are children aged 4-17 years. Thirty-three GPs were connected to the HONEUR network (General Practitioners Research Network Erasmus University Rotterdam) [14]. Recruitment occurred between May 2004 and March 2006. During consultation the GP informed the child and its parents about the study. After receiving written informed consent from the child and/or the parents, a research nurse contacted the child/parents and made an appointment for a visit within one week. All research nurses were aware of the code of conduct for resistance of minors participating in medical research.

### Assessment of selective recruitment

To examine whether our cohort adequately represents children consulting the GP with a new episode of abdominal pain, we retrospectively searched the EMRs of 27 randomly selected participating GPs, for eligible children coded as D01 "generalized abdominal pain", D06 "localized abdominal pain", D11/D70/D73 "gastroenteritis", D12 "constipation" and D93 "IBS". These International Classification of Primary Care (ICPC) codes were chosen because children with these diagnoses are likely to present with abdominal pain [15]. For all children identified, their age and sex were registered. The age of non-participating children was calculated halfway the inclusion period.

### Primary outcome measures

The primary outcome measure is the presence of CAP after 12 months as defined by von Baeyer et al., i.e. occurrence of abdominal pain at least once each month in the past 3 months [16]. In addition, this abdominal pain had to interfere with the child's daily activities; therefore, at least one of the following situations had to be answered with 'yes': Was the abdominal pain severe enough to: 1) stay home from school, terminate or avoid play, 2) take medication for the abdominal pain, or 3) rate the pain intensity as moderate to severe. To obtain data on pain characteristics the age-adjusted 'abdominal pain index' (API) was used [17,18]. In children aged 8-17 years, pain intensity was determined using a numerical rating scale (NRS) ranging from 0 (no pain) to 10 (worst pain possible). In children aged 4-8 years the pain faces scale was used, i.e. 6 faces ranging from happy/no pain (0), to very sad/worst pain imaginable (10) [19]. To fulfill the criterion for pain intensity the pain had to be scored ≥ 3/10 (children aged 8-17 years) or ≥ 4/10 (children aged 4-8 years) on at least 2 of the following 3 questions: "How severe was the pain usually experienced during the past 2 weeks?", "How severe was the worst pain in the past 2 weeks?" and "How severe is the pain right now?". Impairment of daily activities was recorded by parents or child (≥ 9 years) on the Functional Disability Inventory (FDI)[20]. The FDI was designed as a global measure of functional disability for use in research regarding the impact of illness on children's physical and psychosocial functioning in everyday social roles. The FDI consists of 15 items, each scored from 0 ("no trouble") to 4 ("impossible"). The total score is the sum of the responses to the 15 items, with a maximum of 60 points (Table 1).

**Table 1:** Primary outcome measures and potential prognostic factors

	Evaluation (months)
***Primary outcome measures:***	
- Chronic abdominal pain as defined by von Baeyer et al.	**0 3 6 9 12**
- Functional Disability Inventory	**0 3 6 9 12**
	
***Prognostic factors/secondary outcome measures:***	
- Patient characteristics/demographics	**0 3 6 9 12**
- Pain characteristics	**0 3 6 9 12**
- Gastrointestinal symptoms	**0 3 6 9 12**
- Medical history/co-morbidity	**0**
- Negative life events	**0**
- Family history	**0**
- Psychological features	**0 12**
- Illness perception	**0**
- Coping strategies	**0**
- Bullying	**0**
- Healthcare use	**0 3 6 9 12**
- Management by GP	**0 3 6 9 12**
- Laboratory results	**0 12**
- Body mass index	**0 12**

### Baseline measurements

At the inclusion visit (mostly at home) the research nurse measured body weight and height, and collected urine samples. Faeces samples were collected at home on 3 consecutive days in separate baskets, of which one contained a SAF fixative (triple-faeces test), and were sent together in a sealed envelope to the laboratory by regular post [21]. All additional data were collected by standardized questionnaires on abdominal pain, answered by the child and/or parents. Research nurses were trained to give uniform and adequate information about the questionnaires in case either a child or a parent asked for it. Demographic data were collected with a structured questionnaire filled in by the parents or child (≥ 9 years). To obtain data on pain characteristics the age-adjusted 'abdominal pain index' (API) was used [17,18]. Furthermore, the questionnaire yielded the following information: relationship of pain to sleep, location of pain, relationship to meals (worse, better, no effect), associated GI symptoms, associated non-GI symptoms (headache, urinary symptoms, fever), interference with patient's usual activity, family history of GI disorders, and any medications used in the past 2 weeks. Family history was recorded by the parents. Co-morbidity was recorded by the parents or the child (≥ 9 years).

Using a standardized bowel questionnaire, the number of stools, consistency of stools, variability of pattern, blood on stool, and mucus on stool were assessed. Questions were phrased as those normally asked in daily practice and easily comprehensible for the child. Life events were recorded by the parents using a validated Dutch questionnaire with items on 24 life events [22]. Illness coping strategies were recorded by parent and child (≥ 9 years) using the Illness Behavior Encouragement Scale (IBES) [23]. Psychological characteristics were recorded by parents using the age-adjusted CBCL: children aged 4-5 years CBCL 1 12-5 (edition 2000), and children aged 6-17 years CBCL 6-18 (edition 2001) [24]. For children aged 11 years and older, the Youth Self-Report was used [25]. Beliefs about causal factors and illness perception were recorded by the parent or child (≥ 9 years) using 5 structured questions and 1 open question. Healthcare consultations and GP's management were recorded by parent or child (≥ 9 years) using 16 structured questions and 1 open question. Filling in these baseline questionnaires took (on average) about 1-1.5 h for the parent and/or the child.

### Laboratory analyses

The GP was unaware of the results of urine and faeces analyses performed for the study, determined his/her own treatment, and was free to do what he/she thought was best for the child's well-being. This guaranteed customary medical treatment. Analyses were done in the Microbiological Laboratory of the Erasmus MC and were preserved anonymized. Urinary tract infection (UTI) was defined as >1000 pathogens/ml urine with a sediment of >6 leucocytes and/or ≥ 3 erythrocytes/ml urine. In case of a positive culture without sediment the test was interpreted as no UTI. At the end of follow-up the researchers were given first notice of the results for evaluation and from that moment onward the results were also accessible for the GP. When a child left the study prematurely the GP (with permission from the child or its parents) could request the results before the end of the initial follow-up.

### Follow-up

After 3, 6 and 9 months the inclusion questionnaires on pain characteristics, additional symptoms, medication use, healthcare use, GP management and the functional pain index were sent by post. At the end of the study, or in case of premature quitting, children filled in a concluding questionnaire. At 12 months, urine and faeces samples were collected and the research nurse measured weight and height at a home visit. At this visit all inclusion questionnaires (except for family history, medical history of the child, and co-morbidity) were answered by the child and/or its parents (Table 1). In addition, information on the GP's management of the child during the first 3 months after inclusion, and the final diagnosis, were extracted from the EMRs held by the GPs.

### The diary

Children were asked to fill in an abdominal pain diary during 2 weeks from the moment of inclusion and at 3, 6, 9 and12 months follow-up. In this diary they reported their mood, change in daily activities due to abdominal pain, use of medication due to abdominal pain, use of other 'pain management' strategies, and school absence. In addition, children answered questions about their defecation pattern (number of stools, consistency of stools, variability of pattern, blood on stool, and mucus on stool) and their nutritional habits. The diary was adapted from the headache diary of Pothmann et al. and was modified to cover the topic of the present study [26]. The purpose of this diary was to get insight into the frequency of abdominal pain, the impact of abdominal pain on daily activities, and to evaluate possible relations between abdominal pain, defecation pattern and nutritional habits

### Prognostic factors and secondary outcome measures

The following are considered as possible prognostic factors for CAP after 12 months as defined by von Baeyer et al.: sex, age, educational level of the recording parent and child, baseline severity and duration of pain, concomitant symptoms, history of abdominal surgery, functional disability, negative life events, family history of (non-) GI symptoms, psychological features of the child, and illness perception. Also evaluated are: parental coping strategies (using the IBES questionnaire), bullying, use of healthcare, initial management by GP, BMI and pathogens in urine and faeces (Table 1).

### Sample size

In children without prognostic factors, the prevalence of CAP at 12 months is expected to be 30%. We aim to detect a relative risk (RR) of 2 for CAP at 12 months, i.e. in the presence of a prognostic factor the probability of CAP at 12 months will increase to 60%. Given that a prognostic factor will be present in 10% of the children without CAP, we need to analyze 250 children in order to detect an RR of 2 with a power of 80% and an α of 0.05. If the prevalence of the prognostic factor is <10%, the power to detect this RR will decrease; in that case we remain able to detect higher RRs with equal power. With an expected loss to follow-up of 20%, we need to include 300 children presenting to their GP with a new episode of abdominal pain.

### Statistical analysis

Descriptive statistics are used to describe the data. Means and standard deviations (SD) are presented to summarize continuous variables. The included and non-included eligible children are compared by calculating differences in total participation percentages and participation percentages of specific ICPC codes with corresponding 95% confidence intervals (95%CI). Proportions were compared for continuous variables using Chi-square tests; means were compared using an independent sample t-test. All analyses were performed using SPSS version 15.0 (SPSS Inc, Chic, Ill, USA).

In case of parents answering 'don't know' on family history, we assumed they did not suffer from these ailments. Furthermore, in case ≤ 3/15 items on the FDI were reported as missing we interpreted these as '(0) no trouble'. For all other variables, the number of patients that filled in the questionnaire on that particular item is reported.

## Results

### Study sample

During the 21-month inclusion period (with a mean inclusion period per general practice of 19.3 months) participating GPs recruited 348 children with a new episode of abdominal pain. In total 306 (87.9%) gave written informed consent. Reasons stated by the 42 non-participators were: not interested (n = 13, 31.0%), not meeting inclusion criteria (n = 8, 19.0%), lack of time (n = 6, 14.3%), too much bother (n = 5, 11.9%), too many problems (n = 4, 9.5%), and miscellaneous (n = 6, 14.3%). In one child baseline questionnaires were missing; this child was excluded from further analyses. This resulted in a total of 305 participants with a mean age of 8.30 years (SD ± 2.96), 189 were female (62.0%) and 142 children (46.6%) met Baeyer's criteria for chronic or recurrent abdominal pain (CAP) at inclusion.

### Patient selection

The 27 randomly selected GPs included 213 patients; 149 of them were identified by the ICPC search in the EMRs of the practices, 64 of the included children (30.0%) were not identified, i.e. they were not coded by the GP. No differences were found in age (mean difference -0.53 years; -1.41 to 0.36), sex (OR 1.34; 0.74 to 2.42) and diagnosis by ICPC coding (Chi-statistic 5.2, p = 0.16) between these 149 identified included patients and the 64 included not-identified patients. The search in the EMRs revealed 556 eligible children, of whom 149 (26.8%) participated in the study. It is unknown how many of the non-participating children were not included because of inability to complete the questionnaire, or refusal to give informed consent. The 149 included children were significantly younger than the 407 identified non-participating children; 8.49 versus 9.20 years (0.71; 0.13 to 1.30), respectively. The percentage of children aged ≥ 12 years was 14.8% (n = 22) among participants and 23.1% (n = 94) among non-participants. The overall participation percentage of girls was not significantly higher than that of boys (OR 1.42; 0.97 to 2.09). In both girls and boys mean age of non-participants was slightly higher than that of participants though these differences were not statistically significant. Compared to the total participation percentage of 26.8%, the participation percentages of individual ICPC codes were significantly different. This difference was 11.9% (4.6% to 19.3%) for D01 and -20.0% (-25.7% to -14.4%) for D11/D70/D73. This indicates a relative over-representation of D01 "generalized abdominal pain" and under-representation of D11/D70/D73 "gastroenteritis" (Table 2).

**Table 2:** Proportions of ICPC codes of identified participants versus total sample

International Classification of Primary Care (ICPC)	Patients identified in the EMR	Study participants	Difference in proportions (95%CI)*
All codes	556 (100%)	149 (26.8%)	
Generalized abdominal pain (D01)	222	86 (38.7%)	11.9% (4.6% to 19.3%)
Localized abdominal pain (D06)	120	37 (30.8%)	4.0% (-5.0% to 13.1%)
Constipation (D12)	76	17 (22.4%)	-4.4% (-14.5% to 5.6%)
Gastroenteritis (D11/D70/D73)	133	9 (6.8%)	-20.0% (-25.7% to - 14.4%)
Irritable Bowel Syndrome (D93)	5	0 (0%)	-26.8% (-30.5% to - 23.1%)

* Difference between mean total participation percentage (26.8%) and participation percentage of specific ICPC codes

### Power

The prevalence of most prognostic factors was sufficiently high to detect a RR of 2 for persisting CAP at 12 months with a power of 80%. A history of abdominal surgery, a family history for IBD or Helicobacter pylori infection and faecal pathogens had a prevalence <10%; therefore, the study will have less power to detect small increases in risk for persisting CAP in children with these determinants with equal precision (α 0.05) (data not shown).

## Discussion

We present a one-year follow-up study of a cohort of school-aged children presenting with a new episode of abdominal pain in general practice. At inclusion 46.6% of all children already met Baeyer's criteria for CAP. The included children were significantly younger than the identified non-participating children. Furthermore we found an underrepresentation of children with gastroenteritis and a relative overrepresentation of generalized abdominal pain in our cohort.

Participants were on average 8 months younger than non-participants, and there was a significant over-representation of D01 "generalized abdominal pain" and an under-representation of D11/D70/D73 "gastroenteritis". The mean age of the participants being lower than that of non-participants is probably due to adolescents' lack of willingness to participate. Only D01 "generalized abdominal pain" was age related; its over-representation was less marked in children ≥ 12 years. The selective inclusion of young children might explain the over-representation of "generalized abdominal pain" which is a very non-specific "diagnosis"; in older children the differential diagnosis will be broader and therefore more specific ICPC codes might be used.

The under-representation of "gastroenteritis" may be explained by the fact that children with gastroenteritis will primarily complain of vomiting and diarrhoea rather than of abdominal pain and, thus, did not meet the inclusion criterion of a new episode of abdominal pain. Included children therefore mainly represent school-aged children aged ≤ 12 years with functional abdominal pain; abdominal pain for which no objective evidence can be found for an underlying organic disorder. This is precisely the group for whom data on prognosis and prognostic factors are most needed.

We identified 18 studies evaluating the course of CAP; fourteen studies included children referred to secondary/tertiary care, in 3 studies setting was not reported and one study was population-based [27]. None of these studies followed children seen in general practice. A recent study investigated predictors of persistent gastrointestinal symptoms among new presenters to primary care. This study, however, included adults aged 25-65 years [28]. To gain fuller insight into the prognosis of CAP and the consultation behavior of children with CAP, more studies in general practice are needed.

To our knowledge, this is the first cohort study in primary care to investigate the predictive value of patient characteristics and symptoms for the prognosis of childhood abdominal pain. We expect that the effects of selective recruitment will not cause significant bias, as adjustments will be made in future analyses for age and sex. Our study has sufficient power to analyse most factors currently associated with the prognosis of abdominal pain, although an association between persisting pain and the investigated pathogens can only be detected when the association is strong. Our cohort represents young school-aged children consulting the GP with a new episode of abdominal pain. We are confident that the results will contribute to the current knowledge on abdominal pain in childhood.

## Competing interests

The authors declare that they have no competing interests.

## Authors' contributions

LAAS performed statistical analyses and drafted the manuscript. APJMH participated in study design, data collection and revised the manuscript critically. YvL participated in data cleaning, interpretation of the data and drafting the manuscript. MAB participated in study design and revised the manuscript critically. SMAB participated in study design and revised the manuscript critically. JP participated in study design and revised the manuscript critically. MYB participated in study design and coordination, data interpretation and drafting the manuscript. All authors read and approved the final manuscript.

## Pre-publication history

The pre-publication history for this paper can be accessed here:

http://www.biomedcentral.com/1471-2296/11/27/prepub
